# Acquired Myelodysplasia or Myelodysplastic Syndrome: Clearing the Fog

**DOI:** 10.1155/2013/309637

**Published:** 2013-10-07

**Authors:** Ethan A. Natelson, David Pyatt

**Affiliations:** ^1^Professor of Clinical Medicine, Weill-Cornell Medical School and Director, Transitional Residency Program, Houston Methodist Hospital, 6550 Fannin Street, Suite 1001, Houston, TX 77030, USA; ^2^Summit Toxicology, LLP, 1944 Cedaridge Circle, Superior, CO 80026, USA; ^3^Schools of Pharmacy and Public Health, The University of Colorado, Denver, CO 80026, USA

## Abstract

Myelodysplastic syndromes (MDS) are clonal myeloid disorders characterized by progressive peripheral blood cytopenias associated with ineffective myelopoiesis. They are typically considered neoplasms because of frequent genetic aberrations and patient-limited survival with progression to acute myeloid leukemia (AML) or death related to the consequences of bone marrow failure including infection, hemorrhage, and iron overload. A progression to AML has always been recognized among the myeloproliferative disorders (MPD) but occurs only rarely among those with essential thrombocythemia (ET). Yet, the World Health Organization (WHO) has chosen to apply the designation myeloproliferative neoplasms (MPN), for all MPD but has not similarly recommended that all MDS become the myelodysplastic neoplasms (MDN). This apparent dichotomy may reflect the extremely diverse nature of MDS. Moreover, the term MDS is occasionally inappropriately applied to hematologic disorders associated with acquired morphologic myelodysplastic features which may rather represent potentially reversible hematological responses to immune-mediated factors, nutritional deficiency states, and disordered myelopoietic responses to various pharmaceutical, herbal, or other potentially myelotoxic compounds. We emphasize the clinical settings, and the histopathologic features, of such AMD that should trigger a search for a reversible underlying condition that may be nonneoplastic and not MDS.

## 1. Introduction

 Despite advances in cytogenetic and flow cytometric analyses, aberrant cellular morphology, as identified in the peripheral blood and bone marrow, remains the defining feature leading to a clinical diagnosis of myelodysplastic syndrome (MDS). Certain laboratory values such as blood cell count and cell volume measurements are accurate and reproducible, and the results are not open to dispute, as is the presence of particular unique and obvious morphologic findings such as the presence of acquired Pelger-Huët granulocytes and tear-drop erythrocytes in the peripheral blood or large numbers of ringed sideroblasts or increased numbers of myeloblasts in the bone marrow. Other observations such as reduced mature myeloid cell cytoplasmic granulation and the presence of dimorphic erythrocyte or dysmorphic megakaryocytic populations are more subtle. However, what constitutes a significant variation from normal in each of the three major cell lines in the bone marrow remains very observer dependent. Unfortunately, we are only occasionally but usefully reminded that not all clear-cut examples of acquired and persistent myelodysplasia represent MDS or neoplasia [1, 2]. 

 The difficulty with morphology, alone, in establishing a diagnosis of MDS is evident in the evolution of the current World Health Organization [WHO] classification system for MDS with respect to the acquired refractory sideroblastic disorders. Germing and associates suggested that careful morphological review allowed some separation within the initial MDS classification system of those individuals with acquired idiopathic sideroblastic anemia (AISA) who were more likely to have an illness that would terminate in AML from those who might not have a neoplastic or preleukemic condition. They separated 232 individuals with MDS associated with ringed sideroblasts into two groups, one without significant myelodysplastic features among nonerythroid bone marrow cells and the other exhibiting such dyspoiesis among multiple cell lines. The 38% with selective erythroid aberrations and the 62% with a more multilineage dysplasia, respectively, exhibited different clinical courses, frequency of cytogenetic defects, and survival patterns [3]. Earlier, other authors had also proposed that AISA was not a uniform illness and that some affected individuals actually had a “benign” form of the disorder [4].

 Such an arbitrary distinction among those with a sideroblastic MDS was subsequently adopted in the WHO MDS classification as refractory anemia with ringed sideroblasts (RARS) and refractory cytopenia with multi-lineage dysplasia and ringed sideroblasts (RCMD-RS). However, a uniform concordance with this dual classification among experts in the field seemed hopelessly lacking. In Pavia, Italy, experienced hematopathologists classified only 28% of 60 such MDS cases with ringed sideroblasts as RCMD-RS while their colleagues in Dusseldorf, Germany, opined that 76% of their 119 patients with MDS and ringed sideroblasts fell into this category [5]. To solve this dilemma of lack of agreement in classification, the WHO simply eliminated the category of RCMD-RS with the publication of their 2008 fascicle. The result was that the diagnosis of RARS seems to be disappearing as fewer hematopathologists seem to be willing to commit to a unilineage myelodysplasia in their interpretation of bone marrow morphology. Thus, RARS, which once amounted to more than 10% of all MDS, despite the original inclusion of the myeloproliferative disease, chronic myelomonocytic leukemia [CMML] as MDS now only accounted for 1.1% of all MDS in a recently analyzed group of 611 cases [6]. Nevertheless, many clinical hematologists still recognize RARS as a specific entity and wonder why the morphology-based separation between the two ends of a bell-shaped curve, which may represent perhaps the single most distinctive form of MDS, was even attempted [7]. 

**Figure 1:**
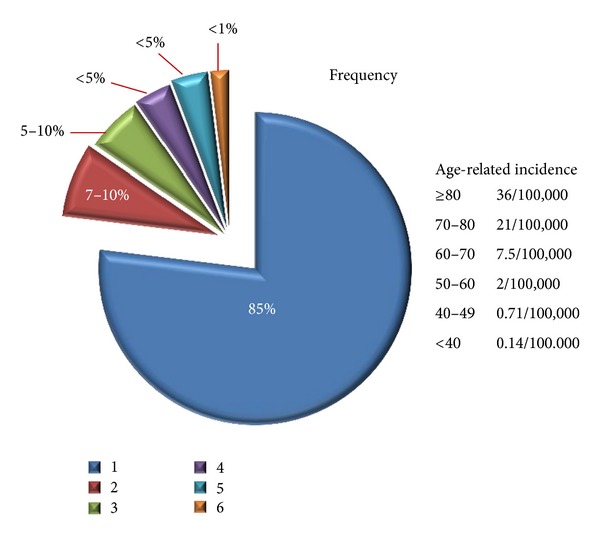
Age-related incidence of MDS and frequency estimates of MDS subsets. (1) *De novo *MDS (primary): estimates are 85% of all MDS. About 45–50% manifest cytogenetic abnormalities evident by standard analysis with recurring examples being del (5q), del (7q), del (20q), and trisomy 8. (2) Secondary MDS: estimates are 7–12% of all MDS with >80% relating to prior therapy with mutagenic chemicals and/or radiation (therapy-related, t-MDS). Chromosome aberrations are present in >90%, particularly involving chromosomes 5 and 7, and often associated with complex cytogenetics and poor prognosis. (3) Cigarette smoking is strongly associated with MDS/AML and directly relates with the total amount smoked and current smoking at diagnosis. (4) Subsets of MDS characterized by ringed sideroblasts may have different causation from other MDS syndromes. (5) Perhaps 5–10% of individuals with MDS as defined only by morphologic aberrations and cytopenias and with clinical features suggesting autoimmune disease may respond favorably to immunosuppressive therapy. (6) Occupational/environmental chemical exposures are thought to cause <1% of all MDS with benzene-related disease some fraction of this amount.

 Current and suggested future MDS classifications seem to focus primarily on survival statistics or risk for evolution into AML to complement prognostic scoring systems [8]. Such data are not useful for epidemiological studies searching for the etiology of the initial process or necessarily dictating the therapy of specific types of MDS as advocated and applicable for other complex hematologic disorders such as the non-Hodgkin lymphomas [NHL]. Figure 1 indicates the age-related incidence and an estimated frequency distribution of subsets of MDS that relate with etiologic circumstances or associations rather than survival risk.

 The ability to “see” and report myelodysplasia where none is likely to exist was emphasized in a recent blinded study involving inspection of the bone marrow aspirate slides of 120 healthy prospective bone marrow donors with normal blood counts by four allegedly experienced morphologists [9]. Here, more than 10% of the bone marrow cells were found to exhibit myelodysplasia involving one cell line in 37% of this cohort, among two cell lines in 31%, and among all three cell lines in 6.5% of these individuals, none of whom would be reasonably expected to have either myelodysplasia or MDS. Such observations speak to the inherent weakness of morphologic interpretation in current WHO MDS classification systems despite attempts at clarification [10].

 When a clinical hematologist is confronted by a bone marrow study interpreted as MDS, typically, that diagnosis has been made with a little difficulty and is thought likely to be correct [10]. However, both the presence and the absence of certain supportive clinical observations and laboratory findings and the disease setting should give a pause for thought and avoid tacit acceptance of the diagnosis without consideration and exclusion of other potential entities. As a prominent medical educator, and Master of the American College of Physicians (MACP), cautions us, “*From time to time almost all of us practice what I call elephant medicine. Like elephants in the circus ring—the trunk of one holding on to the tail of the other—we plod mindlessly along, following without question the diagnoses and decisions of our colleagues*” [11]. Some of these cautionary circumstances and histological observations in relation to a diagnosis of MDS are outlined in the list below, and several combined features may be present in any particular example. Some of these caveats deserve a specific comment, and we include brief, illustrative summaries of three cases that represent AMD but not MDS, in which one of the authors (Ethan A. Natelson) was a consultant.

Clinical and laboratory features where AMD may not represent MDS.

1. Young age (<40).
2. Lack of erythrocyte macrocytosis.
3. Lack of cytogenetic aberrations.
4. Presence of ringed sideroblasts.
5. Amegakaryocytic thrombocytopenia.
6. Multiple vacuoles in erythroid and/or myeloid precursor cells.
7. Absence of increased numbers of myeloid blast forms.
8. Prior use of prolonged antibiotic therapy.
9. A history of herbal and/or unregulated alternative medication use.
10. Evidence of systemic or cutaneous autoimmune conditions preceding myelodysplasia.
11. Human immunodeficiency virus (HIV) infection.

## 2. Results

### 2.1. Patient Age

The incidence of MDS increases dramatically with advancing age, and MDS is uncommon among individuals younger than 50 years, where it accounts for only 6-7% of all MDS and much less when the diagnosis is restricted to a *de novo* presentation [12–14]. In a recent MDS cohort without such restriction, only 3 of 70 patients were younger than 40 years [15]. The overall incidence of MDS in the United States per 100,000 individuals aged 70–79 is 20.94 and in those less than 40 years only 0.14 [16]. Other consistently noted specific features of this younger group with MDS include the infrequency of a hypocellular bone marrow and the infrequency of RARS morphology, the latter observation particularly striking among children [13, 17]. Thus, when evaluating a young adult with a presumptive diagnosis of MDS associated with large numbers of ringed sideroblasts, normal cytogenetics, and no increase in blast forms, one should always seek to identify an alternative diagnosis.

### 2.2. Absence of Erythrocyte Macrocytosis

Mean corpuscular red blood cell volume (MCV) measurements above 100 fl occur in less than 2% of the general population but are nearly a universal finding among patients with MDS, where the MCV is often above 106 fl and may be as high as 140 fl, particularly among those individuals with RARS. Thus, to be referred an individual with suspected MDS featuring a sideroblastic anemia associated with a normal MCV, and in the absence of chromosomal aberrations, and where vitamin B-12 and folic acid deficiency have been excluded, certain alternate maladies should immediately come to mind. For example, despite the impressive numbers of ringed sideroblasts, both lead and arsenic poisoning typically are associated with an MCV in the normal range [18, 19]. Copper deficiency, which may be a consequence of bariatric surgery, and excessive use of zinc, also causes an anemia with a refractory sideroblastic bone marrow appearance but usually with a normal or even reduced MCV, unless other vitamin deficiencies are present [20–23]. Copper deficiency may also result in multiple cytoplasmic vacuoles among the bone marrow erythroid and myeloid precursors, which is not a feature of MDS [21, 23]. Additionally, copper deficiency has been associated with the presence of multiple clumps of hematogones (clusters of lymphoid precursors) in the bone marrow which should not, but may be, confused with increased numbers of blast cells suggesting MDS/AML [21].

### 2.3. Sideroblastic Anemia

As discussed in the previous section, reversible sideroblastic anemias may occur in a number of circumstances including prolonged use of certain antibiotics such as chloramphenicol, linezolid, pyrazinamide, isoniazid, rifampin, and some tetracyclines [24, 25]. In this group the myelodysplasia that results may from time to time also be associated with the acquired or pseudo Pelger-Huët anomaly of abnormal granulocyte nuclear segmentation. The presence of such atypical mature but bilobed granulocytes in the peripheral blood is often sought as a diagnostic marker of MDS [26–29]. Perhaps not well appreciated, a number of drugs may also cause the pseudo-Pelger-Huët nuclear anomaly. It is increasingly noted in organ transplant patients receiving immunosuppressive medications and, in particular, tacrolimus and mycophenolate mofetil [28, 29]. Thus, it is not surprising that the combined aberrant morphology of drug-induced sideroblastic anemia with pseudo Pelger-Huët cells might well be misinterpreted as clear-cut evidence of MDS [30]. For example, in a large cohort of patients with MDS, a statistically significant increased incidence of MDS was reported to be associated both with tuberculosis and with the use of herbal or traditional medications [31]. However, large and well-controlled epidemiological studies show no association of MDS with tuberculosis or other infectious diseases [32]. Moreover, lead, arsenic, and mercury poisoning as described in the previous section have been reported as contaminants of unregulated traditional medications and may cause refractory sideroblastic anemias. Thus, the observed, statistically significant association of MDS with tuberculosis and herbal medications in this study likely represents inclusion of AMD cases in the analysis [31]. Later commented on further, one of the first examples of effective treatment of apparent MDS with immunosuppressive therapy was described in a patient with RARS [33].

### 2.4. Absence of Cytogenetic Aberrations

Certainly, examples of MDS without numerical or molecular cytogenetic aberrations occur but are uncommon, particularly in secondary or therapy-related MDS. As cytogenetic analysis has become more sophisticated and accompanied by specific and comprehensive fluorescent *in situ *hybridization (FISH) probe panels and the developing nucleotide polymorphism array technology, the frequency of a normal cytogenetic result in MDS with a cellular bone marrow has been greatly reduced [34]. Particularly in the proper setting, the lack of a demonstrable cytogenetic abnormality should always increase suspicion that MDS may not be the correct diagnosis despite persistent morphologically evident myelodysplasia. Conversely, 14 recurring cytogenetic aberrations that allow a presumptive diagnosis of MDS to be established despite inconclusive cellular morphology are outlined in a recent review of MDS classification [35].

### 2.5. Absence of an Increase in Myeloid Blast Forms

Whether a bone marrow demonstrating 10% or more myeloblasts should still be considered a form of MDS, rather than AML, has always been a very controversial issue [2]. Certainly, major cancer centers have often advocated treating such patients with acute myeloid leukemia [AML] chemotherapy protocols, and the associated hematological illness is clearly a neoplasm and would not be confused with AMD. Currently, diagnostic blast cell percentages in MDS/AML continue to be enumerated by morphology, but advances in flow cytometry are likely to ultimately prove a more accurate measure and to define other cellular features which may improve specificity in the diagnosis of MDS [34, 36].

### 2.6. An Associated or Preceding Autoimmune Disorder

Many have commented on the association of autoimmune disease preceding or coincident to the development of hematological abnormalities consistent with MDS, and this association has also been well documented by epidemiological studies [32, 33, 37–43] Some suggest that as many as 10% of all MDS represent an immune-related illness [37]. Particularly, profound myelodysplasia consistent with MDS may occur following the onset of certain cutaneous disorders such as Sweet syndrome, bullous pemphagoid, several forms of vasculitis, with certain rheumatoid disorders including relapsing polychondritis, polyneuropathy, and inflammatory bowel disease [37, 40–43]. Remission from both the skin disease and the associated MDS may occur simultaneously with effective immune-mediated therapy, as in our Case 2.

### 2.7. Acquired Amegakaryocytic Thrombocytopenia with Cellular Bone Marrow

While thrombocytopenia in the setting of MDS is common and may be associated with reduced numbers and dysmyelopoietic-appearing megakaryocytes, acquired amegakaryocytic thrombocytopenia with a cellular bone marrow is unusual in MDS but has been described in association with lupus erythematosis and thymoma and as an immune consequence of several disorders [44]. It may also respond favorably to immunosuppressive therapy as in our Case 1. Again, in concert with other clinical and laboratory manifestations of what appears to be MDS, amegakaryocytic thrombocytopenia may be a clue to search for an alternate diagnosis.

### 2.8. Vacuolization of Bone Marrow Erythroid and Myeloid Precursor Cells

Years ago, extensive vacuole formation, particularly in the cytoplasm and even the nucleus of bone marrow erythroid precursors, was a hallmark of chloramphenicol toxicity. Today, a similar process has been noted with toxicity to the antibiotics linozolid and certain tetracyclines. This finding has been also described with arsenic poisoning and copper deficiency where it may affect both myeloid and erythroid cells and is well known as a consequence of alcohol-induced hematological toxicity along with ringed sideroblasts [21, 23, 24, 45]. Importantly, it is highly unlikely to be the primary or sole feature of MDS, as in our Case 3, and its presence should immediately suggest a medication review and a search for a possible recent bone marrow toxic exposure.

**Case 1**

A 29-year-old white woman who worked in the home as a computer specialist had been scheduled for her initial visit at a bone marrow transplant center in order to discuss, and then undergo, allogeneic stem cell transplantation for progressive cytopenias consequent to MDS. Features of two prior bone marrow studies allowed multiple hematopathologists and her hematologist to concur with the diagnosis of MDS. However, fearful of the complications of bone marrow transplantation, she had been referred to yet another hematologic consultation by her primary physicians not for a review of the diagnosis but simply to convince her of the need for urgent transplantation. Several months earlier she first began to experience easy bruising and was found to have a hemoglobin concentration of 11.0 gm/dL with a platelet count of 63,000/cu mm. She was thought to have idiopathic thrombocytopenic purpura [ITP], but, despite therapy with corticosteroids and Win-Rho SD immune globulin, she became progressively pancytopenic and had required blood transfusions. Numerous laboratory studies including antinuclear antibody, Coombs' tests, serum vitamin B-12, folate, serum copper and blood lead levels, parvovirus, hepatitis A, B, and C panels, and HIV titres gave normal results as did CT scans of the chest and abdomen. Her CBC now showed a hemoglobin concentration of 6.6 gm/dL with an MCV of 107 fl. Her total leukocyte count was 2,100/cu mm with a neutropenia, and she had a platelet count of 8,000/cu mm.

Her peripheral blood film showed macrocytosis, tear-drop, and other abnormally shaped erythrocytes. The two bone marrow specimens were similar, with about 70% cellularity and with only rare megakaryocytes. Erythropoiesis was increased in activity and extremely dyspoietic. Iron stains showed numerous ringed sideroblasts. Granulocytopoiesis was reduced in activity with mild dysplastic changes. Blast cells were not increased. Routine cytogenetic studies had twice given normal (*diploid*) results. For the first time, in evaluating the cause for her myelodysplasia and cytopenias, the history was elicited that she had suffered a spontaneous miscarriage shortly before she began to experience excessive bruising. The event was known to her primary care physician, who attributed no significance to it, and her referring hematologist was unaware of it. Now, suspecting a diagnosis of pregnancy-induced pancytopenia based upon the unusual spectrum of peripheral blood and bone marrow findings and prior clinical experience, she was treated with a course of anti-thymocyte globulin and oral danazol, as described in a previous publication [46]. Within a few months she fully recovered a normal blood count and was advised not to again become pregnant.

However, a year later, asymptomatic and with a normal CBC, she again became pregnant. Her pancytopenia and macrocytosis soon reappeared within the first trimester, and the cytopenias were more profound than on her first presentation. The platelet count was now 2,000/cu mm with a total leukocyte count of 2,200/cu mm with a neutropenia, and she again required blood transfusions. She declined the suggestion by her high-risk obstetrician of a therapeutic termination of pregnancy. She received low-dose prednisone and cyclosporine and required frequent platelet and red blood cell transfusions throughout the pregnancy but uneventfully came to term with only continuous severe bruising. A healthy female infant with a normal blood count was delivered by C-section. She then received oral mycophenolate mofetil and prednisone and within 4 months achieved a normal blood count, and all medications were discontinued. She later underwent bilateral tubal ligation and remained well with a normal CBC, five years later.

Pregnancy-induced pancytopenia is a rare disorder that may occur with the first pregnancy where it may be mild and spontaneously remit to reappear with subsequent pregnancies, often with increasing severity. In this regard, it is similar to the rare syndrome of circulating inhibitor of factor VIII induced by pregnancy, and the infant is not affected by the process. While the peripheral blood and bone marrow, at first glance, seem typical of MDS, the constellation of teardrop erythrocytes in the peripheral blood, increased bone marrow cellularity with erythroid hyperplasia, amegakaryocytic thrombocytopenia, and ringed sideroblasts in a young woman in the proper clinical setting should suggest the correct diagnosis and not MDS.

In a recent publication calling for all MDS to be renamed as the myelodysplastic neoplasms (MDN), the authors' comment was “*⋯The risk of non-MDS patients being treated erroneously as having MDS is relatively low*” [47]. Certainly, this patient might argue that point, particularly if she were required to convince her insurance company that she did not have a malignancy and underwent an unnecessary allogeneic stem cell transplant procedure!

**Case 2**

A 52-year-old white attorney with type II diabetes mellitus developed a macular pruritic rash on his upper arms typical of Sweet syndrome that, over several months, became generalized and excoriated. Various topical preparations, antihistamines, and pulse steroid doses were not helpful. He experienced a 20-pound weight loss and developed intermittent fever. He became anemic and thrombocytopenic. He underwent upper and lower endoscopies which gave normal results as did CT scans of the chest and abdomen. His hemoglobin concentration was 10 gm/dL with an MCV of 105 fl. The total leukocyte count was 7,000/cu mm with a monocytosis and the platelet count 102,000/cu mm. A bone marrow study revealed an 80% cellular marrow with an increase in megakaryocytes and trilineage dysplasia, particularly evident in erythroid elements. Iron stores were normal without ringed sideroblasts. Blast forms were not increased. His bone marrow was interpreted by the hematopathology department as consistent with MDS. Cytogenetic studies gave normal results.

Considering an immune-related AMD, he received a trial of oral prednisone and 6-mercaptopurine, and his hemoglobin improved to 14.0 gm/dL with a normal MCV and a platelet count of 232,000/cu mm. His skin rash faded but reappeared when the immunosuppressive drugs were tapered and discontinued. His platelet count then fell to 44,000/cu mm and his hemoglobin concentration to 8.6 gm/dL. Prednisone was resumed, and mycophenolate mofetil substituted for the 6-mercaptopurine. Now, 3 years later and receiving only 500 mg of mycophenolate mofetil daily, aside from his usual diabetic medications, his skin rash is well controlled, his CBC is normal with a normal MCV, and he is asymptomatic.

Sweet syndrome has been described in MDS, AML, and NHL but often abruptly affects younger individuals with no evident underlying neoplasm and typically responds well to immunosuppressive therapy [41, 42]. Its presence preceding the presumptive cytopathological diagnosis of MDS without cytogenetic aberrations or an increase in myeloblasts and a cellular bone marrow should suggest that MDS may not be the correct diagnosis of AMD and prompt a trial of immunosuppressive therapy.

**Case 3**

A 56-year-old white man, who was employed as mechanic, and was a heavy smoker, developed severe, generalized, and disabling arthritis, thought by his rheumatologists to represent seronegative rheumatoid arthritis. His blood counts and chemistries initially gave normal results. Over a period of 2 years he received courses of at least five different nonsteroidal anti-inflammatory agents. When this therapy proved ineffective, he received oral methotrexate for several months, along with continued anti-inflammatory medications. He then underwent a bone marrow study because over this two-year period his hemoglobin concentration had declined from 14.3 gm/dL to 12.6 gm/dL and his total leukocyte count from 7,000/cu mm to 3,300/cu mm, with a neutropenia. His platelet count remained in the normal range. The striking morphological abnormality in the bone marrow was extensive vacuolization, particularly among the myeloid cell precursors. There was no increase in blast forms, and cytogenetic studies gave normal (*diploid*) results. He was thought to have MDS but required no specific therapy for this illness. Over time, his anemia spontaneously improved, but he remained with mild neutropenia and with a normal platelet count until he died 5 years later from complications of pulmonary fibrosis, a well-known association with the rheumatoid diseases [48]. It was alleged in a legal action, later dropped, that on the basis of his bone marrow study he had MDS, caused by his exposure, years earlier, to benzene, as it was contained in diesel and other fuels he worked with. As discussed, vacuolization of bone marrow precursors is not a primary feature of MDS, and his mild and nonprogressive cytopenias were likely treatment-related.

## 3. Discussion

Despite our current knowledge and growing laboratory expertise, whether a prolonged hematopoietic myelodysplastic reaction associated with cytopenias but without a distinct pattern of clonal cytogenetic aberration or increase in blast forms is actually MDS, as we currently employ this term for risk assessment, may remain uncertain. Such an unusual circumstance is presented by the work of Irons and associates in China, involving individuals with alleged major exposures to pure benzene and who manifest unusual and complex bone marrow histology frequently associated with bone marrow hypoplasia, multilineage dysplasia, atypical eosinophilia, and phagocytic histiocytes, among other features [6, 49, 50]. A similar type of general hypocellular bone marrow histology with eosinophilia was described among a large Brazilian cohort with chronic benzene poisoning, but a long-term followup to determine recovery or progression to MDS, AML, aplastic anemia, or other illness was never described, and cytogenetic studies apparently were not done [51]. This cohort was not described as having MDS and, one would assume, rather thought to have a form of chronic myelosuppression, capable of recovery, but possibly with later risk for onset of AML, as described in Aksoy's work in Turkey [52].

 In Irons and associates two initial reports, this distinctive hematological toxicity to benzene was described simply with morphologic descriptive terms such as dysplasia and dyserythropoiesis [49, 50]. In their most recent publication, these cases are classified as MDS. However, more than twice as many subjects were placed in the MDS-U (*unclassifiable*) category as their control subjects with MDS [6]. MDS-U is a seldom-employed subset of MDS, and, with such few cases, a separate survival risk assessment is not available [36]. Nevertheless, 80% of Irons and associates' cases were alive at 60 months, with a continuing flat survival analysis at the time of publication compared with the nonbenzene exposed MDS group with a steadily declining survival, circa 25%, at that follow-up interval. The latter observation would be consistent with the usual survival statistics in MDS and far superior to survival characteristics in typical chemical-induced MDS [53]. Moreover, Irons and associates' cohort, overall, had a lesser frequency of chromosome aberrations than routinely seen in *de novo* MDS and far less frequently than described in chemical-induced or secondary MDS, even among other individuals with their hematological illness claimed to be related to solvent and petrochemical exposures and exhibiting high frequencies of aberrations in chromosomes 5 and 7 and the 5 q-syndrome. 

 Other medical groups in China, where major and prolonged benzene exposures are still possible, have classified their alleged examples of benzene-induced hematotoxicity in a different manner than Irons and associates. In one report the authors separate 41 such patients as representing either aplastic anemia, pancytopenia (*presumably signifying chronic myelosuppression*), or MDS, with the latter diagnosis only accounting for 4 members of this cohort [54]. They emphasize that many of these subjects responded favorably to removal from continuing benzene exposure along with the use of androgens and immunosuppressive medications. As Irons and associates observed, their few cases with MDS also had excellent long-term survival [6, 54]. 

 A caveat is that cytogenetic studies in China for MDS/AML have shown a differing frequency of particular aberrations than among Western patients, with an increased frequency of trisomy 8 and a very reduced incidence of the 5q-syndrome as well as a marked increase in t (15; 17) acute promyelocytic leukemia [55, 56]. Another confounding factor might be the frequent use of traditional or herbal preparations among this population [31]. We would not suggest arbitrarily classifying this seemingly unique AMD as something other than MDS, but we prefer to await a longer followup on the index patients and to see if others observe this unique type of hematologic toxicity and outcome among different ethnic populations with exposures to similar levels of benzene.

 Olnes and Sloan have demonstrated that, in selected patients with MDS, a significant number may respond favorably to immunosuppressive therapy and observed a complete remission in 18% of 31 evaluable patients, including one manifesting a small clone with trisomy 13 [40]. This patient received treatment with alemtuzumab, a monoclonal antibody directed against the T cell marker, CD 52, which has also produced favorable responses in nonneoplastic associated autoimmune hemolytic anemia as well as in B cell neoplasms such as chronic lymphocytic anemia [57]. They also suggest that the ideal candidates for a trial of immunosuppressive therapy are younger individuals with low risk forms of MDS but did not feel that the degree of marrow cellularity was a predictor of favorable response. The trisomy 13 was a concern but, by analogy, small clones of trisomy 15 associated with pancytopenia may spontaneously regress associated with improvement in the pancytopenia, and a small aberrant clone does not always imply preleukemic syndromes [43, 58]. Thus, the presence of small clones of certain cytogenetic aberrations should not eliminate consideration of a trial of immunosuppressive therapy, although the ideal drug combination to employ is not established. Patients with trisomy 8, even with hypocellular bone marrows, may respond favorably to immunosuppressive therapy possibly for reasons apart from classical mechanisms in autoimmune disorders [59]. Many other agents and drug combinations have been effective in immune-related therapy of MDS and include tissue necrosis factor-*α* (TNF-*α*) inhibitors and compounds that may bind with T-lymphocyte CD receptors and modify the expression of interleukin-2 and other cytokine production [34, 60].

 In MDS with hypocellular bone marrow, this circumstance may be difficult to clearly separate from aplastic anemia where favorable responses to drugs such as anti-thymocyte globulin [ATG] are often seen. Nevertheless, we believe that a young individual with MDS, regardless of bone marrow cellularity and normal cytogenetics, particularly when associated with morphologic features such as sideroblastic anemia or amegakaryocytic thrombocytopenia occurring in the clinical setting of autoimmune illness, are with high likelihood of achieving a favorable response to immunosuppressive medications. As emphasized, it seems critical to initiate therapy with immunosuppressive regimens early in the course of MDS in order to favorably impact the long-term result [60]. This may seem difficult to recommend in a patient with low-risk MDS who is not particularly symptomatic. Nevertheless, delaying such therapy may contribute to the adverse immune response by facilitating the continuing presentation of apoptotic cell-generated autoantigens to T-lymphocytes.

 Bone marrow flow cytometry (FC) analysis may be useful in an attempt to separate MDS from other causes of persistent cytopenias, including forms of hypoplastic anemia. Its application can identify specific aberrations in both immature and maturing cell compartments among the hematopoietic cell lineages but, as yet, it is not reliable as a single parameter to segregate MDS as a specific diagnosis [61]. There may be no universal FC marker pattern to prove MDS in all cases because of the heterogenous nature of the disorder. The information obtained by FC is most useful with analysis of lineage fidelity or infidelity among the immature myeloid progenitor cells and least useful with disorders of megakaryocyte lineage. It is particularly difficult to use FC to isolate MDS from nonneoplastic conditions characterized by cytopenias and a normal basic cytogenetic pattern. Nevertheless, guidelines are being established to describe a FC panel as normal, suggestive of MDS, or consistent with MDS [61]. Repeated FC assessments are recommended, particularly in low-risk and/or inconclusive MDS patients, to document changes and monitor the course of the disease.

 The WHO MDS classification seems to favor a parallel association with risk stratification [62]. Since the usual survival risk of MDS-U is claimed to be similar to that in the RCMD subset, and, since only 6% of 2032 patients with MDS were classified as MDS-U, a recent recommendation is that the MDS-U category simply be eliminated from the WHO MDS classification scheme and combined with RCMD [63]. This approach would recapitulate the fate of the former RARS-MD MDS subset. Since the category of MDS-U requires <10% cellular dysplasia, it would then be difficult to find a suitable home in this proposed WHO MDS system for many of these subjects classified as MDS by Irons and associates [6]. 

 Whether or not all MDS should be considered neoplasms and whether or not Irons and associates' experience with persistent cytopenias among heavily benzene-exposed individuals should be classified as MDS and as neoplasms are just some of the unanswered questions in this field. There is considerable information that immune dysregulation plays an important role in the onset and progression of MDS both confounding a certain confirmation of the diagnosis and its uniform classification as a malignancy. The most straight-forward evidence of this influence is the favorable therapeutic response seen among certain patients with MDS, as defined by current diagnostic criteria, who are treated with medications with immunosuppressive activity. The frequency of long-term control of the illness with this form of therapy is uncertain, but as many as 30% of all MDS cases exhibit some hematologic improvement when treated with a variety of immunosuppressive agents [60]. What remains unclear are the underlying mechanisms by which the immune system may regulate myeloid cell development and either promote the disease or modulate progressive failure of normal hematopoiesis.

 In low-risk forms of MDS, based upon current stratification schemes, among those with bone marrow hypocellularity, increased apoptosis of bone marrow cells is an established pathogenic mechanism resulting in ineffective hematopoiesis. Several compelling lines of evidence suggest that disruption of the differentiation of hematopoietic progenitor cells and their increased apoptosis is immunologically mediated [64–70]. Moreover, in many examples of low-risk MDS, inflammatory cytokines appear to be driving the disordered immune response, including the observed increase in apoptosis. Many investigators have reported aberrations in the TNF-*α* pathway in immunosuppressive-responsive examples of MDS [66, 69, 71–77]. TNF-*α* plays a fundamental role in mediating apoptotic pathways among multiple cell types, and an increased production or deregulated role could result in the observed bone marrow pathology. At least in low-risk MDS, there is also support for the involvement of various components of the innate immunity pathway such as natural killer [NK] cells and macrophages [76, 78–80]. The increased frequency of NK and activated macrophages in the MDS bone marrow may increase the elaboration of cytokines such as gamma interferon and TNF-*α* and other proapoptotic cytokines, which, in turn, results in the observed increase in apoptosis and peripheral blood cytopenias [76, 81, 82]. NK-cell mediated cytotoxicity toward aberrant cell precursors occurs in many low-risk examples of MDS [78]. Macrophages are believed to play important roles in bone marrow regulation and hematopoiesis via contact with adhesion molecules [80, 83]. Apoptosis occurs in low-risk MDS cases among both clonal and nonclonal progenitor cells, but this feature begins to decrease as the MDS evolves toward a more malignant phenotype [84].

 The increase in apoptosis observed in low-risk MDS is thought to be a key step in the pathology of this disorder. However, there may be additional modifying immunological events in play that collectively result in the observed morphological dyspoiesis. Dysplasia-associated antigens released by degenerating cells that are processed by antigen-presenting cells may initiate an adaptive immune response that may influence the course of the illness [76]. Evidence in support of active involvement of the immune system includes an increase in the number and activation of various effector cells, promoting an autoimmune environment. There is a clear relationship between autoimmunity and some forms of bone marrow failure and dysplasia [76, 85]. The autoimmune-promoting environment in example of low-risk MDS includes increased levels of proapoptotic cytokines, increased numbers of helper T cells, altered humeral immunity, and reduced levels of regulatory T cells [Treg]. Treg cells are CD-4 and Fox-P3 positive and are known to play an important role in immune surveillance and self-tolerance and in suppressing autoimmunity [76, 86]. It has been suggested that reduced numbers of Treg cells and other immunological changes may result in T cell-mediated inhibition of normal hematopoiesis [76, 78–80].

 TNF-*α* mediated apoptosis may be a highly relevant mechanism to promoting bone marrow dysplasia reported following excessive benzene exposure [48–50, 86]. Previous reports indicate that benzene metabolites and TNF-*α* act synergistically to induce apoptosis in CD-34+, bone marrow progenitor cells [87–89]. Further, epidemiologic evidence suggests that polymorphisms in TNF-*α*, resulting in deregulated TNF-*α* production, increase susceptibility to benzene-induced hematopoietic toxicity [49, 87, 90]. There is also limited evidence that immunosuppressive therapy provides therapeutic benefit in the treatment of what has been described as benzene-related MDS, as this illness appears to be similar to benzene-induced aplastic anemia, which also tends to respond favorably to immunosuppressive therapy [53].

 This pattern of immune dysregulation that may be modified by immunosuppressive therapy is not characteristic of more advanced or high-risk cases of MDS. Here, there are increased numbers of regulatory cells, which dampen any type of favorable response to immunosuppressive therapy. This may, in turn, allow for a damaged or dysplastic clone to escape from immune surveillance and progress to a more aggressive expression of the disease [88, 89]. Further, in high-risk examples of MDS, pathological changes include apoptosis resistance, which could allow secondary genetic abnormalities to appear and stimulate an increase in cellular proliferation, thus providing potential growth advances for the aberrant clone and resulting in a much higher propensity for evolution into AML [91].

 Immunosuppressive therapy seems to be most effective when applied early in those with low-risk MDS. Many studies have used the combination of anti-thymocyte globulin (ATG) and cyclosporine A (CsA), because of their well-known benefit in forms of aplastic anemia and in the organ transplant field. However, corticosteroids, alemtuzumab, and newer immunosuppressive agents, such as mycofenolate mofetil and TNF-*α* inhibitors, have also been effective in MDS and may be used on a chronic basis, unlike ATG, which is typically administered as a single infusion because of the potential for severe reactions to additional challenges.

 While the fog obscuring the certain diagnosis of MDS is slowly lifting, clinicians must remain alert to the possibility of an alternate disorder in certain cases of AMD. Our therapeutic armamentarium continues to improve along with efforts to define in which examples of MDS and in which clinical settings they are most likely to initiate a favorable response. While it is clear that immunosuppressive therapy may be beneficial in a subset of individuals with MDS, the most effective types of immunosuppressive regimens and the duration of such therapy remain to be determined. Moreover, such favorable response does not necessarily imply a causative autoimmune illness or exclude a neoplasm.
